# Umbilical Cord Blood-Derived Products in Autoimmune Systemic Syndromes with Severe Dryness: A Pilot Study

**DOI:** 10.3390/medicina60111764

**Published:** 2024-10-28

**Authors:** Rosario Foti, Marco Zeppieri, Roberta Foti, Ylenia Dal Bosco, Riccardo Foti, Antonino Maniaci, Fabiana D’Esposito, Giuseppe Gagliano, Caterina Gagliano

**Affiliations:** 1 Division of Rheumatology, A.O.U. “Policlinico-San Marco”, 95123 Catania, Italy; rosfoti5@gmail.com (R.F.);; 2 Department of Ophthalmology, University Hospital of Udine, p.le S. Maria della Misericordia 15, 33100 Udine, Italy; 3 Plastic and Reconstructive Surgery, Department of Surgical Sciences, University of Rome Tor Vergata, 00133 Rome, Italy; 4 Department of Medicine and Surgery, University of Enna “Kore”, 94100 Enna, Italy; 5 Imperial College Ophthalmic Research Group (ICORG) Unit, Imperial College, 153-173 Marylebone Rd, London NW1 5QH, UK; 6 Department of Neurosciences, Reproductive Sciences and Dentistry, University of Naples Federico II, Via Pansini 5, 80131 Napoli, Italy; 7 Faculty of Medicine and Surgery, University of Catania, 95100 Catania, Italy; 8 Mediterranean Foundation “G.B. Morgagni”, 95125 Catania, Italy

**Keywords:** umbilical cord blood serum, ocular surface disorders, autoimmune systemic syndromes, Sjogren’s syndrome, systemic sclerosis, dryness, Schirmer test, xerophthalmia

## Abstract

*Background and Objectives*: Human umbilical cord blood serum (HUCBS) stands out as a potent adjunct to conventional therapies for ocular surface disorders (OSDs) caused by, among many, autoimmune systemic syndromes. By expediting ocular surface regeneration and fostering epithelial integrity, HUCBS not only enhances subjective patient experiences but also improves objective clinical indicators. This makes it particularly useful in patients with corneal ulcers through ocular surface regeneration and anti-inflammatory activity. This study aims to explore the efficacy of HUCBS in patients who had previously received other treatments unsuccessfully. *Materials and Methods*: This study was a prospective, non-comparative, interventional case series study involving 49 patients (30 females and 19 males) aged 15–82 years with severe OSDs who were unresponsive to standard treatments. The study was conducted at the San Marco Hospital, Catania, Italy. Patients were categorized into four groups based on the etiology of their severe OSDs: Group I consisted of twenty four patients with filamentary keratitis and corneal ulcers associated with rheumatologic diseases such as Sjogren’s syndrome and systemic sclerosis; Group II comprised thirteen patients with graft-versus-host disease; Group III consisted of nine patients with corneal neurotrophic ulcers; and Group IV included three patients with Steven–Johnson syndrome. The outcomes were evaluated before and after treatment using the following assessments: OSDI (Ocular Surface Disease Index) and SANDE (Symptom Assessment in Dry Eye) questionnaires, VAS (Visual Analog Scale), Slit Lamp Examination, Esthesiometry, Lissamine Green Staining, NIBUT (Non-Invasive Break-Up Time), BUT (Break-Up Time), Fluorescein Staining with Photography and Oxford Classification, The Schirmer Test, Best-Corrected Visual Acuity (BCVA), and Meibography. *Results*: We observed a significant improvement in the outcomes from the SANDE, VAS, and OSDI questionnaires, The Schirmer Test, BUT, BCVA, and Oxford Classification, after treatment with UCBS. Clinical variables, such as corneal inflammation, conjunctivalization, corneal neovascularization, and pain, were also considered individually. Nevertheless, pain and inflammation reduced markedly over time until complete healing was achieved in all cases. *Conclusions*: Our pilot study highlights the substantial efficacy of HUCBS in patients with systemic autoimmune diseases who have shown inadequate responses to prior treatments for dry eye. This underscores the need for further comprehensive investigations in this field.

## 1. Introduction

Human umbilical cord blood serum (HUCBS) is derived from the acellular portion of umbilical cord blood collected immediately after birth. The collection is non-invasive, utilizing a natural byproduct of childbirth, and it can be processed and stored with minimal degradation of its bioactive components [1]. This serum has an abundance of various growth factors, cytokines, and anti-inflammatory proteins, making it an effective treatment option for ocular surface disorders (OSDs) [2].

Recent research has increasingly supported the use of HUCBS in OSDs, particularly for patients who do not respond to standard treatments [3]. HUCBS has been shown to significantly speed up the healing of the ocular surface, strengthen the epithelial barrier, and reduce inflammation [4].

HUCBS reduces inflammation through its rich content of bioactive molecules, including growth factors, cytokines, and anti-inflammatory proteins [5]. It contains anti-inflammatory cytokines such as interleukin-10 (IL-10) and transforming growth factor-beta (TGF-β), which help to control the immune response by suppressing pro-inflammatory cytokines such as IL-1, IL-6, and TNF-α [6]. Growth factors such as epidermal growth factor (EGF), hepatocyte growth factor (HGF), and fibroblast growth factor (FGF) promote cellular repair and regeneration, helping to restore the integrity of the ocular surface and further reducing inflammation [7]. The serum’s antioxidant properties also help to reduce oxidative stress, a major contributor to inflammation, by neutralizing reactive oxygen species (ROS) [8]. Additionally, recent studies have shown that HUCBS supports epithelial healing, reduces immune cell infiltration, and prevents apoptosis of epithelial cells, all of which contribute to reducing inflammation and creating a favorable environment for tissue repair [9].

Corneal ulcers represent a sign of severe OSD and are frequently seen in patients with autoimmune systemic disorders. This study investigates the efficacy of UCBS in the treatment of corneal ulcers and other OSDs to assess the wider applicability of this therapy. Recent research has also highlighted the effectiveness of HUCBS in treating corneal ulcers, especially those linked to systemic autoimmune diseases such as systemic sclerosis and Sjögren’s syndrome [10]. Systemic sclerosis is a chronic connective tissue disease that leads to fibrosis of the skin and internal organs, including the eyes [11]. This condition often causes severe dry eye syndrome, which can result in persistent corneal ulcers and significant eye discomfort [12]. Studies suggest that dry eye syndrome in systemic sclerosis patients is closely related to the severity and duration of the disease, with major tear film dysfunction observed due to meibomian gland disease and increased tear osmolarity, making the management of this condition more challenging [13].

Similarly, Sjögren’s syndrome, an autoimmune disorder that primarily affects the body’s moisture-producing glands, leads to severe eye complications, including chronic dry eye and corneal damage [14]. The insufficient tear production and ongoing inflammation of the ocular surface in these patients are linked to complex processes involving both innate and adaptive immune responses [15]. Using HUCBS in these cases has shown not only a reduction in symptoms but also better clinical outcomes, demonstrating its potential as a valuable therapeutic option [16].

The regenerative effects of HUCBS, combined with its ability to modify the inflammatory environment of the ocular surface [17], are particularly beneficial in patients with autoimmune conditions, where chronic inflammation and impaired healing are common issues [18].

The principal benefit of umbilical cord blood-derived products is the abundance of anti-inflammatory cytokines, including interleukin-10 and TGF-β, as well as growth factors such as EGF and FGF, which facilitate ocular surface regeneration. These solutions provide a non-invasive, biologically active therapeutic alternative that surpasses the use of artificial tears and traditional anti-inflammatory medications by targeting both inflammation and tissue repair. Nonetheless, constraints include the fluctuation in concentrations of bioactive components and the logistical difficulties associated with procuring and standardizing these items. These difficulties are alleviated through regulated processes which guarantee a constant quality and efficacy during processing.

Although TGF-β is typically acknowledged for its anti-inflammatory characteristics, it is crucial to understand that its function is “context-dependent”; TGF-β can display anti-inflammatory and pro-inflammatory actions, contingent upon the surrounding microenvironment and the signaling pathways it engages with. In ocular surface problems, TGF-β mitigates inflammation by downregulating the pro-inflammatory cytokines; yet, it may also induce fibrosis and facilitate pathological tissue remodeling in chronic situations. The dual function of TGF-β is well described across multiple tissues, where excessive TGF-β signaling may result in fibrosis, exemplified by disorders such as corneal scarring. Consequently, although the growth factors and cytokines present in umbilical cord blood products, such as TGF-β, may enhance healing and diminish inflammation, it is essential to evaluate their context-dependent effects. To alleviate the possible disadvantages, a rigorous regulation of the concentration of these bioactive compounds during processing is necessary. Furthermore, individualized therapy strategies, considering the patient’s unique pathology and disease stage, may enhance the therapeutic efficacy of these medications. Future research should seek to clarify the circumstances under which TGF-β may transition from an anti-inflammatory to a profibrotic agent in the ocular context.

While artificial tears and lubricants provide temporary relief, they do not address the underlying inflammation or promote healing. Autologous serum eye drops provide nutrients and growth factors but can vary in effectiveness due to differences in patients’ health and blood composition [19]. Anti-inflammatory medications, though effective in reducing inflammation, may have side effects such as increased eye pressure or delayed wound healing [20], and they lack the regenerative properties of HUCBS. Punctal plugs increase tear volume [21] but do not improve tear quality or reduce inflammation, and amniotic membrane transplantation [22], though it promotes healing, is invasive and typically reserved for severe cases.

HUCBS offers a unique combination of benefits: it reduces inflammation, promotes healing, improves tear quality, and provides long-lasting relief with minimal side effects [2]. Its non-invasive nature and effectiveness in treating difficult cases make it a promising alternative or additional therapy for patients with severe OSDs, especially those with corneal ulcers linked to systemic autoimmune diseases such as systemic sclerosis and Sjögren’s syndrome [10]. Given these promising results, there is growing interest in exploring broader uses of HUCBS in refractory cases of OSDs, particularly in patients with complex autoimmune disorders. The use of HUCBS offers a novel and potentially transformative approach to managing these challenging cases, providing hope for better outcomes in patients with limited treatment options.

The aim of this pilot study was to evaluate the efficacy of HUCBS in severe ocular dryness in patients affected by autoimmune systemic syndromes.

## 2. Materials and Methods

This study was a prospective, non-comparative, interventional case series involving 49 patients (30 females and 19 males) aged from 15 to 82 years with severe ocular surface diseases (OSDs) which were unresponsive to standard treatments. The study was conducted at the San Marco Hospital in Catania, Italy, as a result of a collaboration between rheumatologists and ophthalmologists. Patients were categorized into four groups based on the etiology of their severe OSDs as illustrated in Table 1. Although neurotrophic corneal ulcers are not classified as autoimmune diseases, their inclusion in this study aimed to evaluate the effectiveness of umbilical cord blood-derived products across a wide range of severe ocular surface disorders, irrespective of their cause. The results for this cohort are delineated independently from those with an autoimmune etiology.

The outcomes were evaluated both before and after treatment, using a comprehensive range of clinical and symptomatic assessments:

- Ocular Surface Disease Index (OSDI) and Symptom Assessment in Dry Eye (SANDE) questionnaires: utilized to assess the severity of symptoms related to ocular surface diseases and dry eye.
- Visual Analog Scale (VAS): used to evaluate the perceived pain or discomfort.
- Slit Lamp Examination: performed for the clinical observation of the ocular surface and adjacent structures.
- Esthesiometry: used to assess corneal sensitivity.
- Lissamine Green Staining (LGS): a diagnostic test to highlight damage to the ocular surface.
- Non-Invasive Break-Up Time (NIBUT) and Break-Up Time (BUT): measurements indicated the stability of the tear film.
- Fluorescein Staining with Photography and Oxford Classification: employed to evaluate corneal surface damage.
- The Schirmer Test: a test to measure tear production.
- Best-Corrected Visual Acuity (BCVA): assessed to determine the best possible visual acuity with correction.
- Meibography: an imaging technique used to visualize the Meibomian glands, which are crucial for maintaining a stable tear film.

This study aimed to evaluate these parameters before and after the intervention to assess the effectiveness of the treatment administered to patients with severe ocular surface diseases. Patients were monitored at 7, 14, 24, and 70 days after the commencement of treatment to evaluate the critical outcome metrics, including OSDI, SANDE, VAS, The Schirmer test, BCVA, and Fluorescein Staining. These temporal markers facilitated the assessment of both immediate and prolonged treatment effects.

The umbilical cord blood-derived products were retrieved after obstetric data collection (gestational age of the mother, sex and birth weight of the newborn, duration of the labor, Apgar scores of 9 or 10, and the mode of delivery). All the steps from recruitment to processing and registration of the cord blood were performed according to the guidelines provided by Italian Regulations. The serum generated from umbilical cord blood was obtained from healthy post-childbirth donors, processed under sterile circumstances, and stored at −80 °C to maintain its bioactive constituents. The manufacture complied with worldwide requirements for blood product processing, ensuring the retention of the growth factors and cytokines which are vital for therapeutic effectiveness.

This study used cord blood units which were not suitable for hematopoietic stem cell transplantation. The cord blood units were sent to the processing facility at the Sciacca Cord Blood Bank, Sciacca, Italy, for further preparation procedures. The units underwent a series of checks and tests to establish the blood characteristics and their suitability for preservation as blood components for therapeutic use. The cord blood eye drop solution was prepared under sterile conditions. Each dose of platelet lysate produced from the umbilical cord blood was standardized for platelets per microliter. This concentration was established according to the cord blood bank procedures, guaranteeing a uniform treatment efficacy across all patients.

Collected from healthy donors and subsequently processed through centrifugation using an innovative platelet-rich plasma (PRP) preparation kit in a streamlined, single-step procedure, the resulting platelet lysate was then formulated into single-dose eye drops and administered to the patients who were instructed to apply the drops six times daily.

The quality of the eye drops was evaluated based on sterility, endotoxin levels, and platelet concentration, in conjunction with decontamination techniques validated by the Cord Blood Bank. The quality control techniques guaranteed that the eye drops were devoid of microbiological contamination and preserved a uniform bioactive profile.

This study’s outcomes were rigorously assessed before and after the intervention using a variety of clinical and patient-reported measures. These included the Ocular Surface Disease Index (OSDI) and the Symptom Assessment in Dry Eye (SANDE) questionnaires, Visual Analog Scale (VAS) scores, Slit Lamp Examination, Esthesiometry, Lissamine Green Staining, Non-Invasive Break-Up Time (NIBUT) and standard Break-Up Time (BUT), Fluorescein Staining with Digital Photography and Oxford Classification, The Schirmer Test, Best-Corrected Visual Acuity (BCVA), and Meibography.

In Group III, which consisted of patients with corneal ulcers, additional metrics were recorded at each evaluation point, more specifically the size of the ulcer and the relative reduction in size compared to the baseline measurement. Overall, the patients received a total of 90 kits of platelet lysate eye drops. In addition to the primary outcomes, other clinical variables such as corneal inflammation, conjunctivalization, corneal neovascularization, and pain were also individually evaluated to assess the therapeutic impact of the treatment.

All participants provided written informed consent before enrollment onto this study.

This study was conducted as stated by the Declaration of Helsinki. All patients provided written informed consent to the use of their anonymized data for research purposes and scientific publications. The Umbilical Cord Blood Platelet Lysate Eye drop therapy fell within the essential levels of care recommended by Italian Law, which ensures that the procedures controlled by the Ministry of Health are followed. The Institutional Review Board of the San Marco Hospital did not require a trial registration number or approval for this type of routine clinical care.

Data were analyzed using descriptive statistics, with mean values and standard deviations (SD) calculated for all variables. A linear regression analysis was performed to evaluate the correlation between the Break-Up Time (BUT) and treatment duration. Differences in the VAS and SANDE scores over time were assessed using a repeated measures ANOVA, with post-hoc pairwise comparisons conducted where appropriate. A *p*-value < 0.05 was considered to be statistically significant.

## 3. Results

In this study, we observed a significant improvement in the outcomes measured by the SANDE, VAS, and OSDI questionnaires, as well as by the Schirmer Test, BUT, BCVA, and Oxford classification after treatment with HUCBS. These findings indicated a substantial reduction in the subjective symptoms and objective clinical signs associated with ocular surface disease following HUCBS therapy. Significant advances in clinical indicators, including Fluorescein Staining, The Schirmer Test scores, Best-Corrected Visual Acuity (BCVA), and Meibography, were reported following therapy, alongside the improvements in subjective measurements.

Our investigation revealed continuous enhancements in many of the clinical indicators after the use of human umbilical cord blood serum (HUCBS) therapy. The Schirmer Test scores, which evaluate tear production, demonstrated a considerable rise from an average baseline of roughly 3 mm/5 min at pre-treatment to over 10 mm/5 min at post-treatment, indicating a notable improvement in tear production and ocular surface hydration. Likewise, the Best-Corrected Visual Acuity (BCVA) was enhanced in the majority of patients, with visual acuity improvements of at least one line on the Snellen chart observed in 78% of participants. This indicated that the therapy not only diminished ocular surface inflammation but also enhanced visual results, presumably due to the regeneration of the corneal epithelium and the restoration of tear film stability. Furthermore, the Meibography demonstrated a decrease in meibomian gland dropout in multiple patients, hence reinforcing the therapeutic efficacy of HUCBS in enhancing the tear film quality and stability. Fluorescein Staining, utilized to evaluate corneal damage, exhibited a significant reduction in corneal staining intensity following therapy, with the majority of patients transitioning from a Grade 3 or 4 corneal staining to a Grade 0 or 1 at the last follow-up. The decrease in staining with fluorescein underscored the ability of HUCBS to facilitate corneal epithelium repair and diminish surface injury. The enhancements, corroborated by subjective evaluations such as the Ocular Surface Disease Index (OSDI) and the Symptom Assessment in Dry Eye (SANDE) questionnaires, highlighted the many advantages of HUCBS in alleviating both the clinical and symptomatic challenges of severe ocular surface illnesses.

Clinical variables, such as corneal inflammation, conjunctivalization, corneal neovascularization, and pain, were also assessed individually. Notably, both pain and inflammation showed a marked and progressive reduction over time, ultimately leading to complete healing in all cases. This suggests that UCBS is an effective treatment option for reducing inflammation and alleviating pain in ocular surface disease.

The outcomes of the SANDE and VAS questionnaires are presented concurrently, demonstrating notable enhancements in subjective symptoms across all groups. Collectively, these indicators offer an extensive perspective on patient-reported outcomes subsequent to UCBS treatment.

**Figure 1:**
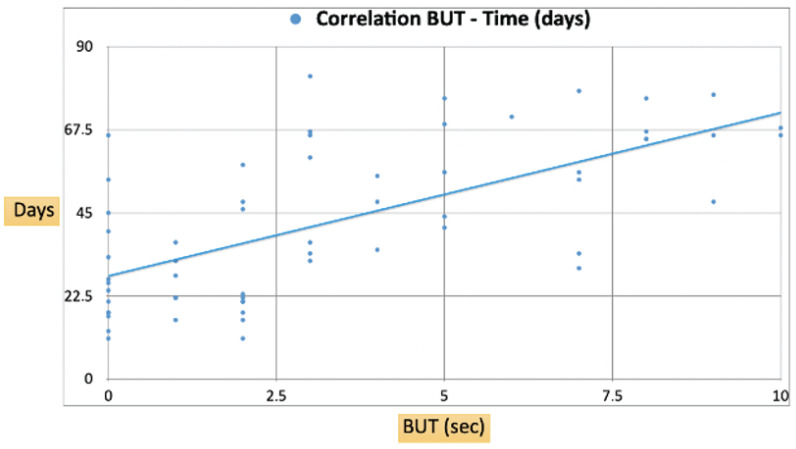
Correlation of BUT–Time (days).

In detail, we observed a progressive increase in the BUT as treatment days advanced as shown in Figure 1. We found a positive correlation between the two variables, suggesting that the tear film stability, as indicated by a longer BUT, improved over time following HUCBS administration. Specifically, the regression line indicated that for each additional day of treatment, the BUT increased by approximately 4.6 s.

Data points at the beginning of the treatment showed a BUT clustered around 1–3 s on the initial days, with notable improvements over the course of treatment. By day 70–80, the majority of patients achieved a BUT exceeding 6 s, with some even reaching up to 9–10 s, indicating significant enhancements in the tear film stability.

**Figure 2:**
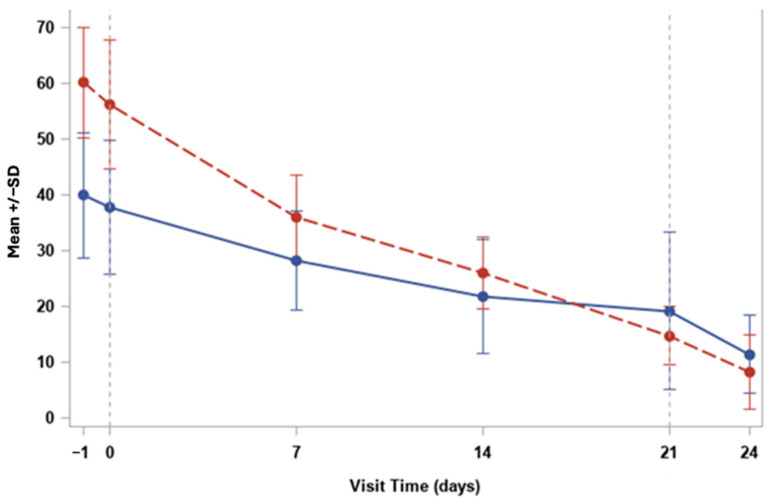
Trend for VAS severity (red) and frequency (blue).

Moreover, at the baseline (Day 0), both VAS severity and VAS frequency scores were elevated in our cohort, with mean values of approximately 65 for severity and 50 for frequency as shown in Figure 2. Over time, both parameters showed a progressive decrease, indicating a reduction in both the intensity and frequency of symptoms.

By Day 7, there was a marked reduction in both scores, with severity decreasing to around 45 and frequency to approximately 30. This trend continued, with both values converging and further decreasing by Day 24 to levels close to 10, highlighting the sustained improvement in symptom relief.

The steady decline in VAS severity suggested a significant reduction in the subjective discomfort reported by the patients, while the decrease in VAS frequency indicated fewer occurrences of symptoms over time.

This reduction in both the severity and frequency of symptoms reinforced the overall efficacy of the HUCBS treatment in alleviating ocular surface disease symptoms in patients with systemic autoimmune conditions.

**Figure 3:**
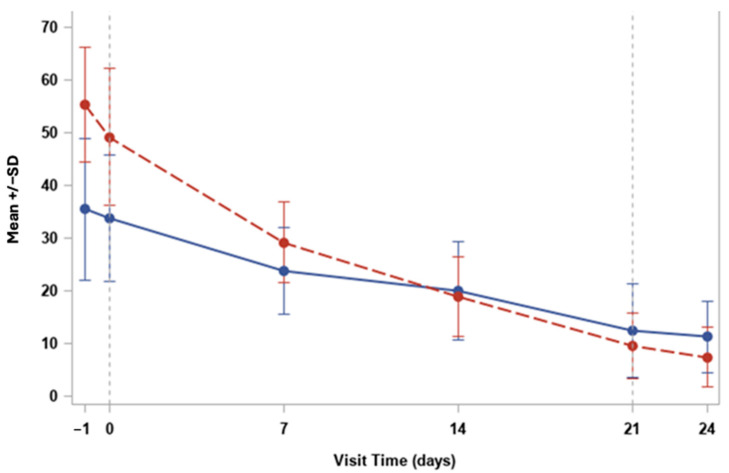
Trend for SANDE severity (red) and frequency (blue).

Finally, at the baseline (Day 0), the mean SANDE severity score was approximately 65, while the SANDE frequency score was around 50, indicating high levels of symptom severity and frequency among the patients as shown in Figure 3. Over the course of the study, both the severity and frequency scores decreased progressively, reflecting the beneficial effects of the HUCBS treatment.

By Day 7, the SANDE severity score dropped significantly to around 40, and the frequency score decreased to 30. The decreasing trend continued consistently across the subsequent time points, with both severity and frequency reaching mean values close to 10–15 by Day 24.

The SANDE severity showed a sharper initial decline, while the SANDE frequency showed a more gradual reduction over time. By Day 21 there was a balanced improvement in both the intensity and the occurrence of symptoms. Furthermore, the application of HUCBS in this study showed a favorable safety profile, with no significant adverse effects reported. This is particularly noteworthy given the challenges associated with other treatment modalities.

## 4. Discussion

The results from this pilot study clearly indicate the therapeutic potential of human umbilical cord blood serum (HUCBS) in managing severe ocular surface diseases (OSDs), particularly among patients with systemic autoimmune conditions such as Sjögren’s syndrome and systemic sclerosis. The substantial improvements in the clinical outcomes, including reductions in pain, inflammation, and corneal damage, underscore the efficacy of HUCBS as a treatment option, particularly in cases where conventional therapies have been unsuccessful. These findings are consistent with the recent literature, which increasingly recognizes the benefits of HUCBS in ocular treatments, particularly due to its anti-inflammatory, regenerative, and healing-promoting properties [2,3,10].

The distinct composition of HUCBS, enriched with growth factors, cytokines, and anti-inflammatory proteins, is likely to be the basis for its effectiveness. Previous research has demonstrated that these bioactive molecules play critical roles in modulating the inflammatory environment, promoting epithelial healing, and restoring the integrity of the ocular surface [4,5]. The observed reduction in inflammation and improvement in tear quality in this study align with the mechanistic actions of HUCBS, which include the suppression of pro-inflammatory cytokines such as IL-1, IL-6, and TNF-α, and the promotion of cellular repair through factors such as EGF, HGF, and FGF [6,7].

Recent studies have also highlighted the effectiveness of HUCBS in treating acute ocular injuries, such as chemical burns. In such severe cases, HUCBS has been shown to significantly enhance the healing process and reduce inflammation, which are crucial in preventing long-term damage to the ocular surface [23]. For instance, studies have reported an accelerated re-epithelialization and reduced corneal opacity in patients treated with HUCBS following acute chemical injuries. These outcomes are attributed to the serum’s rich composition of growth factors and anti-inflammatory cytokines, which not only promote tissue repair but also mitigate the extensive inflammatory response typically associated with such injuries. Additionally, the use of HUCBS in these acute scenarios provided a protective effect on the ocular surface, minimizing further damage and improving overall visual outcomes.

The success of HUCBS in treating corneal ulcers associated with systemic autoimmune diseases is especially encouraging. These conditions often present with complex and multifactorial pathologies, rendering them difficult to treat with conventional methods [12,15]. Supporting these findings, recent clinical trials have demonstrated the efficacy of blood-derived treatments from allogeneic sources in managing the severe dry eye associated with keratopathy. These studies reported significant improvements in ocular surface health and symptom relief, further underscoring the potential of blood-derived therapies, including HUCBS, as valuable treatment options for severe ocular conditions [24]. The observed improvements in tear film stability, corneal sensitivity, and visual acuity further suggest that HUCBS may not only alleviate symptoms but also improve overall ocular function and the patient’s quality of life.

These findings are further supported by emerging evidence in the literature which suggest that HUCBS may be effective in other challenging scenarios, such as neurotrophic corneal ulcers and ocular complications from graft-versus-host disease [10]. The broad applicability of HUCBS across various etiologies of OSDs suggests that its mechanisms of action may be universally beneficial in conditions characterized by chronic inflammation and impaired healing.

Moreover, our study aligns with the promising findings of a clinical trial [25], which compared the efficacy of autologous serum (AS), allogeneic serum (HS), and umbilical cord serum (CS) eye drops in treating severe dry eye syndrome (DES). The randomized, double-blind trial with 63 patients found that all three sera improved clinical outcomes, assessed by visual acuity, The Schirmer Test, tear Break-Up Time, and ocular staining.

However, while the outcomes of this study are promising, it is important to recognize its limitations. The relatively small sample size and lack of a control group limit the generalizability of the findings. The sample size, while sufficient to demonstrate significant effects, is relatively small, and the lack of a control group limits the ability to fully attribute the observed outcomes to HUCBS alone. Additionally, the non-comparative nature of this study prevents direct comparisons with other treatment modalities. Future studies should aim to address these limitations by incorporating larger, randomized controlled trials that can more rigorously evaluate the efficacy and safety of HUCBS and fully explore the potential of HUCBS in this context. Additionally, investigating the long-term effects of HUCBS and its ability to prevent a recurrence of symptoms would be valuable in determining its role as a standard treatment for autoimmune-related dry eye. Nevertheless, the promising results observed in this study lay the groundwork for further research and suggest that HUCBS could play a transformative role in the treatment of ocular surface diseases, offering new hope for patients with limited therapeutic options.

Finally, the findings from this pilot study strongly suggest that HUCBS is a promising treatment option for patients suffering from severe OSDs related to systemic autoimmune syndromes. The gradual and consistent improvement observed in both the VAS and SANDE scores—representing the severity and frequency of symptoms—demonstrates the significant impact HUCBS has on patient comfort and overall ocular health. Notably, the sharp decrease in VAS severity scores during the first week of treatment highlights the immediate relief that patients experienced, particularly in terms of pain and discomfort, which is often a major challenge in managing severe dry eye conditions. This early response can likely be attributed to the anti-inflammatory and regenerative properties of HUCBS, which is rich in bioactive molecules such as cytokines, growth factors, and anti-inflammatory proteins. These components work together to suppress inflammation, promote tissue repair, and enhance tear film stability, thereby addressing both the symptoms and the underlying causes of ocular surface damage.

As the treatment progressed, the continued decline in the VAS and SANDE frequency scores suggests that the therapeutic effects of HUCBS are not only rapid but also sustained over time. By the final follow-up at Day 24, most patients reported minimal symptoms, indicating that HUCBS provided long-lasting relief from the chronic issues associated with autoimmune-related dry eye. This sustained benefit is crucial, as many conventional treatments tend to offer only short-term symptom alleviation, often without addressing the underlying inflammation and tissue damage. The observed increase in the BUT, which correlated positively with the number of days under treatment, further supports the idea that HUCBS contributes to improving tear film stability, a critical factor in the management of OSDs. The BUT measurement, being a direct indicator of tear film integrity, provides objective evidence of the treatment’s efficacy in restoring the normal functioning of the ocular surface, particularly in patients with complex and refractory conditions.

Moreover, the observed increase in the BUT supports the hypothesis that HUCBS treatment contributes to the regeneration of the ocular surface, reducing tear evaporation and improving tear film stability in patients with autoimmune ocular surface disorders.

In addition, the consistent reduction in standard deviation over time, as illustrated in the error bars of both the VAS and SANDE graphs, suggests that HUCBS therapy not only benefits the majority of patients but also leads to more uniform responses across different individuals. This homogeneity in therapeutic outcomes underscores the reliability of HUCBS as a treatment modality, even in a diverse patient population with varying degrees of disease severity. The absence of adverse effects or complications throughout the study is another important factor to consider. This emphasizes the safety of HUCBS, making it a viable alternative or adjunctive therapy for patients who have not responded well to traditional treatments, such as artificial tears, anti-inflammatory drugs, or more invasive procedures such as punctal plugs or amniotic membrane transplantation.

These findings contribute to the evidence supporting the use of HUCBS in the treatment of severe OSDs, particularly in patients with systemic autoimmune diseases who have not responded to conventional therapies. The ability of HUCBS to reduce inflammation, promote healing, and improve clinical outcomes suggests it may offer a valuable alternative or adjunctive treatment in these challenging cases. Further research is warranted to validate these findings, explore the long-term benefits of HUCBS, and potentially expand its use in larger clinical contexts.

## 5. Conclusions

Our preliminary investigation provides compelling evidence of the significant therapeutic potential of human umbilical cord blood serum (HUCBS) in managing patients with systemic autoimmune diseases who have exhibited an insufficient response to conventional treatments for dry eye syndrome. The marked improvement observed in this cohort emphasizes the promising role of HUCBS as an alternative or adjunctive therapy, particularly for individuals who have exhausted standard therapeutic options with limited success. Given the encouraging outcomes of this pilot study, there is a clear and pressing need to undertake more extensive and rigorous clinical trials to further explore and validate the efficacy, safety, and long-term benefits of HUCBS in a broader patient population. These future studies should also aim to elucidate the underlying mechanisms by which HUCBS exerts its effects, potentially paving the way for more targeted and personalized treatment strategies in the management of refractory dry eye associated with systemic autoimmune conditions.

## Figures and Tables

**Table 1:** Patients’ groups.

Group	Patients	Etiology
Group 1	24	Filamentary keratitis and corneal ulcers associated with rheumatologic diseases (Sjögren’s syndrome, systemic sclerosis)
Group 2	13	ocular graft-versus-host disease
Group 3	9	Neurotrophic corneal ulcers
Group 4	3	Steven-Johnson syndrome

## Data Availability

The original contributions presented in this study are included in the article, and further inquiries can be directed to the corresponding author/s.
